# Supplementary material for: Mutations in PCYT2 disrupt etherlipid biosynthesis and cause a complex hereditary spastic paraplegia
Source: Brain. 2019 Oct 22;142(11):3382–97. doi: 10.1093/brain/awz291 (PMC6821184; doi:10.1093/brain/awz291)
Supplement: awz291_Supplementary_Data [file awz291_supplementary_data.zip › awz291-Suppl_data/Supplementary_Data4.zip!/Supplementary information 3 - fibroblast lipidomics.zip!/Fibroblast lipidomics/Controle vs Patient1/Statistical plots and data/Heatmap sorted on FDR.pdf]

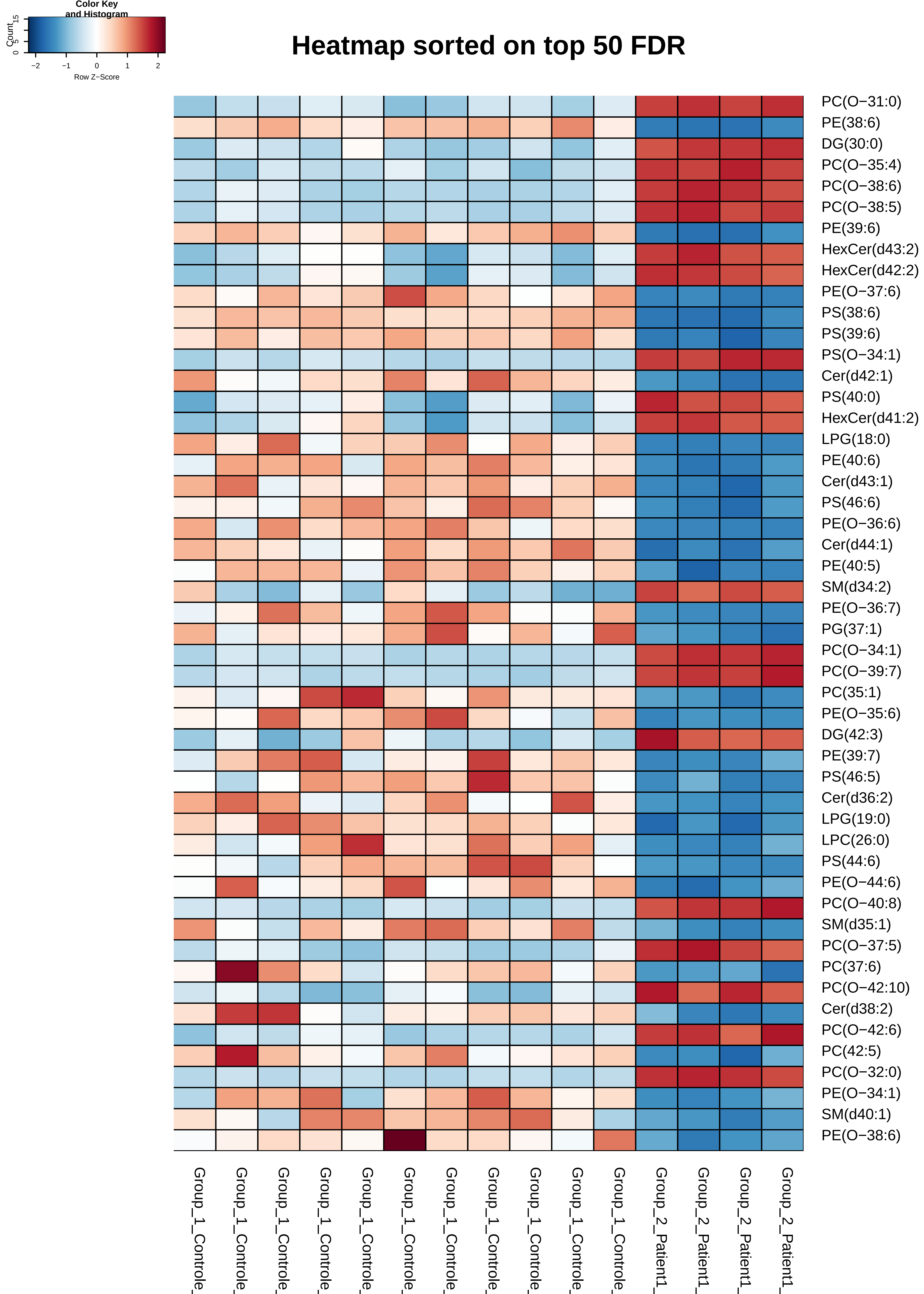
Color Key
and Histogram
Count
0
5
15
−2
−1
0
1
2
Row Z−Score
Heatmap sorted on top 50 FDR
PC(O−31:0)
PE(38:6)
DG(30:0)
PC(O−35:4)
PC(O−38:6)
PC(O−38:5)
PE(39:6)
HexCer(d43:2)
HexCer(d42:2)
PE(O−37:6)
PS(38:6)
PS(39:6)
PS(O−34:1)
Cer(d42:1)
PS(40:0)
HexCer(d41:2)
LPG(18:0)
PE(40:6)
Cer(d43:1)
PS(46:6)
PE(O−36:6)
Cer(d44:1)
PE(40:5)
SM(d34:2)
PE(O−36:7)
PG(37:1)
PC(O−34:1)
PC(O−39:7)
PC(35:1)
PE(O−35:6)
DG(42:3)
PE(39:7)
PS(46:5)
Cer(d36:2)
LPG(19:0)
LPC(26:0)
PS(44:6)
PE(O−44:6)
PC(O−40:8)
SM(d35:1)
PC(O−37:5)
PC(37:6)
PC(O−42:10)
Cer(d38:2)
PC(O−42:6)
PC(42:5)
PC(O−32:0)
PE(O−34:1)
SM(d40:1)
PE(O−38:6)
Group_1_Controle_
Group_1_Controle_
Group_1_Controle_
Group_1_Controle_
Group_1_Controle_
Group_1_Controle_
Group_1_Controle_
Group_1_Controle_
Group_1_Controle_
Group_1_Controle_
Group_1_Controle_
Group_2_Patient1_
Group_2_Patient1_
Group_2_Patient1_
Group_2_Patient1_
